# Deciphering the Assembly of Enveloped Viruses Using Model Lipid Membranes

**DOI:** 10.3390/membranes12050441

**Published:** 2022-04-19

**Authors:** Erwan Brémaud, Cyril Favard, Delphine Muriaux

**Affiliations:** Membrane Domains and Viral Assembly, Montpellier Infectious Diseases Research Institute, CNRS UMR-9004, Université de Montpellier, 1919, Route de Mende, CEDEX, 34293 Montpellier, France; erwan.bremaud@enscm.fr

**Keywords:** viral assembly, biomimetic membranes, membrane proteins, membrane dynamics

## Abstract

The cell plasma membrane is mainly composed of phospholipids, cholesterol and embedded proteins, presenting a complex interface with the environment. It maintains a barrier to control matter fluxes between the cell cytosol and its outer environment. Enveloped viruses are also surrounded by a lipidic membrane derived from the host-cell membrane and acquired while exiting the host cell during the assembly and budding steps of their viral cycle. Thus, model membranes composed of selected lipid mixtures mimicking plasma membrane properties are the tools of choice and were used to decipher the first step in the assembly of enveloped viruses. Amongst these viruses, we choose to report the three most frequently studied viruses responsible for lethal human diseases, i.e., Human Immunodeficiency Type 1 (HIV-1), Influenza A Virus (IAV) and Ebola Virus (EBOV), which assemble at the host-cell plasma membrane. Here, we review how model membranes such as Langmuir monolayers, bicelles, large and small unilamellar vesicles (LUVs and SUVs), supported lipid bilayers (SLBs), tethered-bilayer lipid membranes (tBLM) and giant unilamellar vesicles (GUVs) contribute to the understanding of viral assembly mechanisms and dynamics using biophysical approaches.

## 1. Introduction

The plasma membrane (PM) is a complex cellular interface separating the cytosol from its environment while maintaining exchanges. The PM is mainly composed of phospholipids, cholesterol, sphingo- and glyco-lipids and embedded proteins, sometimes decorated by oligosaccharides and often structured by the underlying cell cytoskeleton [1]. The PM acts as a barrier to control matter fluxes between the cell’s interior and its outer environment; it is a main actor in cell homeostasis. Amongst all the identified viruses, enveloped viruses are surrounded by a lipid membrane derived from the host cell, acquired while budding from the cell after viral particle assembly, i.e., during the late steps in their replication cycle. For example, well known human pathogens such as Human Immunodeficiency Virus Type 1 (HIV-1), Influenza A Virus (IAV) and Ebola Virus (EBOV) assemble at the cell plasma membrane. The molecular interplay occurring during the assembly of viral proteins at the cell membranes is an important process to understand for developing innovative broad-spectrum antiviral strategies. In order to tackle the viral-protein–cellular-lipid interactions occurring during viral assembly, from the molecular to the atomic level, model membranes, made of lipid mixtures mimicking the PM’s properties can be used. These model membranes can be ranked in order of their similarity to the plasma membrane. These include: Langmuir monolayers, bicelles and large and small unilamellar vesicles (LUVs & SUVs), consisting of a first group, in which either one monolayer is absent or the intrinsic curvature is too high; and supported lipid bilayers (SLBs), tethered-bilayer lipid membranes (tBLM) and giant unilamellar vesicles (GUVs), which are better models for the cell plasma membrane. 

**Figure 1:**
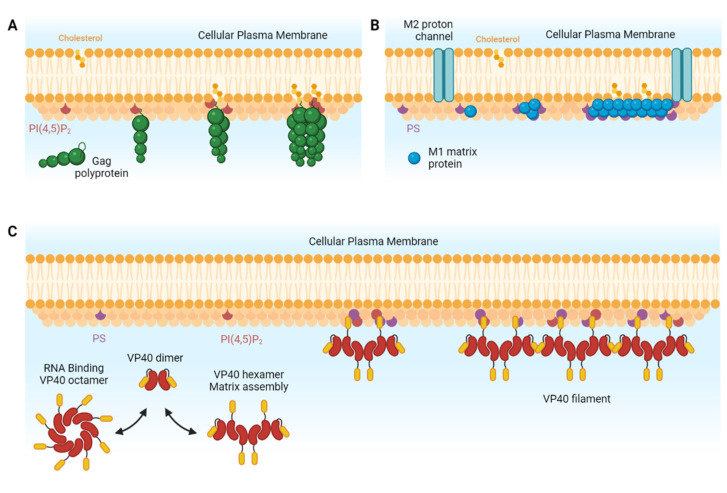
**Molecular steps of virus assembly at the plasma membrane.** (**A**) HIV-1 Gag binds to the membrane via its myristoyl segment insertion and interacts mainly with negatively charged lipids PI(4,5)P_2_ via the HBR region of its MA domain to form lipid domains enriched in PI(4,5)P_2_ and cholesterol. (**B**) IAV M1 interacts with negatively charged PS and clusters cholesterol to form lipid domains. The multimerization was also proposed to be initiated at the location of the M2 transmembrane ion channel. (**C**) EBOV VP40 dimers existing in solution either reorganize in octamers to bind RNA or multimerize in hexamers, which elongate in filaments during assembly.

Viral assembly, which is mainly driven by the self-assembly of viral structural proteins, precedes new particle release from the host cell. In the case of HIV-1, for example, 5 min are required for the viral particle to assemble, whereas budding and particle release occur, on average, 15 min later, independently of the cell type [2,3]. Viral assembly can be seen as a protein polymerization process involving three main steps, namely initiation, elongation and termination. In the case of enveloped virus assembly, initiation is always difficult to define. Here, we define initiation as the nucleation step, i.e., the generation of a nucleus containing a small number of viral and host components, as follows: in the case of HIV-1, structural group-specific antigen (Gag) proteins, the viral RNA genome and host-cell membrane lipids; and, in the case of Influenza, the viral M1 and M2 proteins and the host-cell plasma membrane phosphophatidylserine. Initiation/nucleation requires an energetic barrier to be overcome [4]. From this perspective, the membrane can act as a dimensional catalyzer, which increases the probability of viral protein/viral protein and/or viral protein/genome complex encounters, as well as favoring assembly through entropic effects. In the very first step in membrane assembly, viral proteins are recruited at the membrane, generally via the interaction of the proteins with charged phospholipids. For example, the Matrix (MA) domain of the HIV-1 Gag polyprotein interacts with the phosphoinositol 4,5-bisphosphate, PI(4,5)P_2_ [5,6], (Figure 1A). At the same time, via its nucleocapsid domain, Gag particles also interact with its viral RNA genome, and both phenomena lead to the formation of the nucleus for subsequent Gag protein assembly [7,8]. In the case of IAV, the protein M1, and for EBOV, the protein VP40 are both known to mainly interact with phosphatidylserine (PS) at the inner leaflet of the PM [9,10] (Figure 1B).

Elongation relies on the self-assembly of these structural proteins. It consists in successive first-order processes of monomer addition to the existing nucleus [11,12]. For example, in the case of HIV-1, the Capsid (CA) domain of the Gag polyprotein is responsible for Gag-Gag oligomerization, which corresponds to the elongation step. From this perspective, assembly on the membrane favors correct molecular orientations, helping CA-CA interactions to occur faster [8,13,14]. For Influenza A, the Matrix protein M1 organizes viral assembly at the PM. It was proposed that IAV M1 can only self-assemble at the membrane thanks to its binding to the phosphatidylserine (PS) [15,16], while other studies have proposed that viral proteins such as HA and NA are needed to fulfill assembly [9]. However, recent studies clearly show that the assembly elongation step occurs thanks to the interaction of M1 with the cytoplasmic tail (CT) of the M2 protein, a transmembrane protein ion channel, present at the PM, as the final step in the initiation/nucleation [17] and that HA/NA is not required [16].

The elongation of the virus assembly in EBOV consists in the multimerization of VP40 dimers into an array of dimer-dimer interactions through their C terminal domains (Figure 1C). This elongation step has been shown to be stabilized by the interaction with PI(4,5)P_2_ present in the inner leaflet of the cell plasma membrane [18]. The reader can refer to [10] for more details on the initiation/nucleation and elongation steps of these virus assembly processes.

For all these viruses, structural proteins must contain designated motifs of basic amino acids for either interacting with negatively charged phospholipids to assemble on membranes or recognizing the viral RNA genome for packaging into the particle. The Nucleocapsid (NC) and Matrix (MA) domains of the HIV-1 Gag polyprotein, as well as the Nucleoprotein (NP) of IAV and EBOV, have basic domains and the ability to interact with the viral genomic RNA during packaging, initiating particle assembly. Consequently, enveloped viruses exit host cells with a lipid bilayer pulled out from the PM during particle budding. This viral envelope has two main biological purposes: first, it protects the ribo-nucleocapsid-protein (RNP) complex containing the viral genome from its environment and, second, it embeds enveloping proteins that favor viral entry/fusion thanks to specific interactions with their different host-cell receptors (for a review, see [19,20]). During viral entry, the fusogenic peptide of the viral envelope glycoprotein triggers the fusion of the viral membrane with the cell PM. It liberates the viral genome inside the cell. 

In this review, we will illustrate some of the advances in the understanding of the molecular processes occurring during viral assembly (initiation and elongation) thanks to the use of model membranes. The examples given hereafter are focused on the three human lethal pathogens, namely, HIV-1, IAV and EBOV. 

## 2. An Overview of the Main Types of Model Lipid Membrane

**Figure 2:**
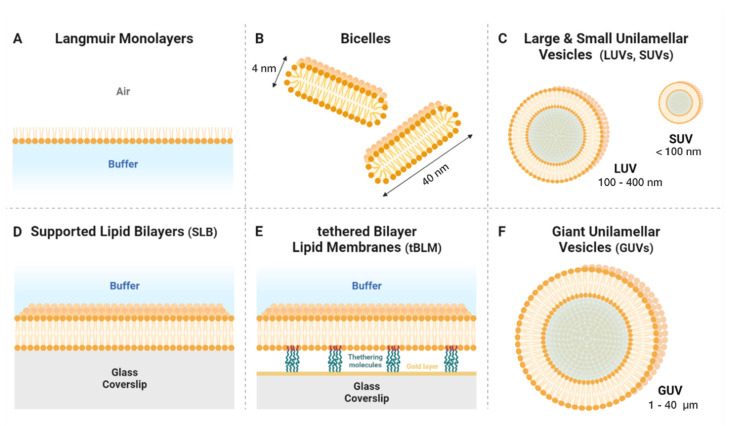
**The different types of model membrane.** (**A**) Langmuir monolayer, which consists in a thin monolayer of lipid at the air/water interface. (**B**) Bicelles are autoassembled droplets of lipids in a polar solvent. Polar heads of lipids are exposed to solvent while apolar acyl chains face each other. (**C**) Large and small unilamellar vesicles are single lipid bilayers separating two polar media vesicles with a diameter of less than 500 nm. (**D**) Supported lipid bilayers (SLBs) are large, flat, single bilayers facing a solid support on one side (mica, glass, etc.) and a liquid buffer on the other side. (**E**) Tethered-bilayer lipid membranes are adaptations of SLBs, displacing the bilayer from the solid support in order, for both faces of the bilayer, to be exposed to the same liquid buffer. (**F**) Giant unilamellar vesicles are the most appropriate membrane model to mimic cell plasma membranes. They consist in a single lipid bilayer separating two (identical or not) liquid media.

The mimicking lipid membranes’ behavior in order to better understand their properties has been attempted for a long time in membrane biology (for a history, see [21]). These lipid-membrane mimics can either be divided into two main classes, i.e., membranes in solution and membranes on surfaces, or, as stated in the introduction, they can be classified with increasing complexity from single monolayers at the air–water interface to micrometric (transversally asymmetric) bilayers in solution. Below, are briefly describe the different types of model membrane through their advantages and disadvantages in terms of mimicking biological lipid membranes (see also Figure 2). 

### 2.1. Langmuir Films

Although originally studied by Franklin and Rayleigh, Langmuir showed that a lipid monolayer can be formed at the air–water interface of a liquid surface. In this monolayer, the lipids are arranged with the headgroups immersed in water and the alkyl chains exposed to air (Figure 2A). Lipid monolayers are typically prepared in a Langmuir film by spreading a lipid organic solvent solution on a water surface, followed by the spontaneous evaporation of the organic solvent. Due to their amphiphilic nature, the lipids will spontaneously locate at the air–water interface, thereby producing the monolayer. One of the main advantages of Langmuir films is that they are easily controlled in terms of physical state (by controlling the surface pressure and area or temperature). They have been widely used to characterize the lipid-phase transition of simple and complex compositions [22]. The possibility of perfectly controlling the lateral pressure imposed on the monolayer invites the study the structural arrangement of lipids by X-ray or neutron reflectivity. However, these types of model membrane are far from the cellular types. 

### 2.2. Micelles and Bicelles 

Micelles can be seen as the soluble version of Langmuir films. Amphiphilic molecules immersed in a hydrophilic buffer have the spontaneous tendency to form micelles, exposing their hydrophilic heads to the solvent while criss-crossing their hydrophobic tails. Inverted micelles can also be spontaneously formed in a hydophobic solvent. While micelles are of little interest for mimicking membranes, bicelles, which represent an intermediate morphology between lipid vesicles and micelles, combine some of the attractive properties of both of these model membrane systems. Bicelles are stabilized at their extremity (Figure 2B) thanks to detergent or detergent-like molecules, such as 3-([3-Cholamidopropyl]dimethylammonio)-2-hydroxy-1-propanesulfonate (CHAPSO) or DiCycloHexyl Phthalate (DCHP). Their ablity to mimick very small soluble bilayers (size <50 nm) makes them the objects of choice for elucidating the structure, using NMR, of membrane-binding proteins [23]. Moreover, they can be oriented in the magnetic field and they have recently been shown to be made of complex mixtures such as SPM, Chol and PL, mimicking the Lo-Ld phase encountered in the so-called “raft” domains [24].

### 2.3. Unilamellar Vesicles

Unilamellar vesicles are small objects (diameter < 400 nm) made of a lipid bilayer (two monolayers) separating two hydrophilic media (see Figure 2C). These types of membrane-mimic, which are also called liposomes, were developed a long time ago and thought to be useful for medical applications [25]. To make them, the solvent in which the lipids are dissolved is first evaporated and the lipid film is gently rehydrated with an aqueous buffer, spontaneously generating multi-lamellar vesicles in the supsension. A series of alternative suspension-freezing/thawing processes is then performed. The next steps can be performed by two different methods: sonication, or extrusion through membranes with different pore sizes (from 30 to 400 nm). Depending on the duration and strength of the sonication, the vesicle size will also vary from 30 to 400 nm. These vesicles are generally named SUVs (small unilamellar vesicles) when their diameters (d) are less than 100 nm, and LUVs (large unilamellar vesicles) when their diameters are >100 nm. They are very simple to prepare and allow all the possible lipid mixtures to be prepared. Howeverer, their small size, meaning a high radius curvature and high membrane tension, classify them as better models for Golgi or Endoplasmic Reticulum (ER) or secretory vesicles than for plasma membranes. 

### 2.4. Supported Lipid Bilayers

One way to avoid this high tension and high radius curvature is to make planar lipid membranes on a solid support. This is the aim of supported lipid bilayers (SLBs), in which the head-groups of the lipids in one leaflet face the support surface, whereas the headgroups of the lipids in the opposite leaflet are exposed to the bulk solvent (Figure 2D). Lipid bilayers have to be deposited on a hydrophilic support surface, such as glass or mica. These SLBs were originally designed by Tamm and McConnell [26]. Two different methods can be applied. The first, Langmuir–Blodgett (LB) or Langmuir–Schaefer (LS) deposition, consists in successively pulling the hydrophilic substrate from the water (buffer) phase of a Langmuir film where the lipids have been deposited in the air phase in order to cover the substrate with a first monolayer and then to dive from the air phase to the water phase through the lipid film, in order to orient the second layer with the polar head exposed to the solvent. The newly made SLB on the support must now stay hydrated. The second method takes advantage of the high curvature radius and membrane tension of the SUVs. A suspension of SUVs is deposited on the hydrophilic/charged surface in order to fully cover its surface after the vesicle adsorption. Due to the high tension, the vesicles tend to spontaneously fuse, leading to the formation of the SLB. This can be facilitated by the addition of divalent cations. Although vesicle fusion is a simpler method than LB/LS, as it only requires the preparation of a vesicle suspension, it typically produces symmetric lipid bilayers, i.e., the two bilayer leaflets exhibit the same composition, whereas LB/LS can be used to produce asymmetric lipid bilayers, since the two leaflets are deposited in two separate steps and can have different lipid compositions. The use of SLBs is often criticized as SLBs exhibit a monolayer in close proximity to the support, which alters the physical properties of the monolayer. To circumvent this limitation, lipid bilayers can also be produced on functionalized surfaces. These SLBs, usually named tethered bilayers, can be produced by functionalizing the support surface with an anchor-lipid, i.e., a modified lipid exhibiting a headgroup that can be chemically bound to the support surface with a spacer (Figure 2E). In order to limit the influence of the support on the physical properties of the lipid bilayer, lipid bilayers can also be produced on supports previously functionalized with a polymer brush, another bilayer or DNA/RNA (for a review, see [27]).

### 2.5. Giant Unilamellar Vesicles 

While the sizes of LUVs and SUVs are measured in tenths to hundreds of nm, giant unilamellar vesicles (GUVs) are measured in µm (from 1 to 100 µm) (Figure 2F). One of the earliest attempts at forming GUVs was the natural swelling method introduced by Reeves and Dowben in 1969 [28]. A lipid solution deposited on a surface is dried to form a lipid film that is then rehydrated and gently stirred to form vesicles. These vesicles are mainly thought to form due to the osmotic pressure driving the aqueous solution in between the stacked lipid bilayers. However, the proportion of GUVs that can be generated using this method is small. A proposed method through which to overcome this drawback involves the gel assisted-formation [29] of GUVs, which provides easy and rapid growth, allowing high yields in the formation process. The second and most famous method is the electroformation method initially proposed by Angelova and Dimitrov. It consists in applying an external electric field during lipid swelling [30]. Platinum wires, indium tin oxide (ITO) and, recently, stainless steel electrodes have been shown to be efficient in producing GUVs by electroformation. GUVs are considered the best cell-plasma-membrane-mimicking models. Their size makes them easy to observe under optical microscope. Recent advances have provided a way to make them transversally asymmetric in order to provide an even better model of the cell plasma membrane. Two main strategies were developed: lipid exchange [31] and hemifusion with SLBs [32]. 

## 3. Artificial Lipid Membranes as Tools for Viral Assembly Research

### 3.1. Langmuir Monolayers: A Fine Tuning System 

A Langmuir monolayer is composed of a single lipid layer at the interface between an aqueous environment and the air. However, unlike cellular PMs, it is very easy to tune its physical parameters, which makes this model system very useful to study protein–lipid interactions that require controlled membrane physical properties (i.e., the lipids’ packing density within the monolayer, or the surface pressure applied to the layer).

The assembly of HIV-1 is mainly driven by viral Gag protein oligomerization. Accessory proteins, such as the viral negative regulatory factor (Nef) protein, could play a secondary role during HIV-1 particle formation, although this role is still debated. Nef was described as promoting Gag membrane localization [33]. Pirrone et al. studied the conformation dependence of myristoylated Nef (myrNef) with lipid and packing density using hydrogen exchange mass spectrometry, or HX MS (see Lexicon for definition) on Langmuir monolayers [34]. They showed that myrNef undergoes a conformational change when the lipid density decreases, turning from a compact conformation adjacent to the membrane into a form in which the N-terminal arm is inserted into the membrane, causing Nef core displacement away from the membrane. This study suggested that, depending on its structure and on the membrane properties, Nef could perform different functions. 

It is firmly established that VP40, the major Ebola Matrix protein, regulates virus assembly and egress at the inner leaflet of the host-cell plasma membrane by recognizing PS [10]. To identify the VP40 amino acids involved in membrane recognition, Adu-Gyamfi et al. generated several VP40 protein mutants and monitored their capacity to penetrate Langmuir monolayers by measuring changes in the monolayers’ lateral pressure. These findings exhibited the involvement of a particular region in the C-terminus domain of VP40 in PM localization, but also in VP40 self-assembly and particle egress [35]. As such measures require fine pressure control over the lipids, Langmuir monolayers are the most appropriate model system to use.

### 3.2. Using Bicelles to Elucidate the Molecular Structures of Viral Proteins on Membranes

**Figure 3:**
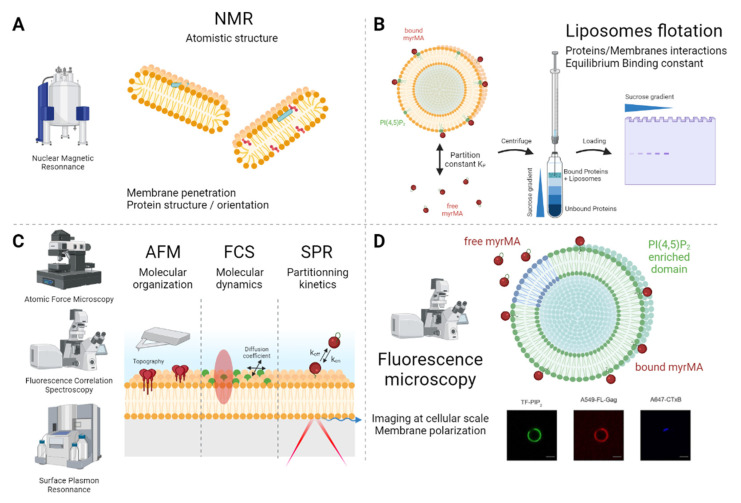
**Methods and techniques for viral assembly study in several membrane model systems.** (**A**) Bicelles are widely used models for protein structural analysis by NMR. (**B**) Liposome flotation unveils partition constant of a protein at a membrane’s surface using LUVs or SUVs. (**C**) Using the SLB planar system, AFM gives topographic information at a molecular level, FCS enables study of molecular dynamics and SPR is a way of studying binding properties. (**D**) GUVs are close in size and shape to cells, and one can, for instance, image protein–-lipid co-localizations at a cellular scale.

Bicelles represent a relatively small, minimal system of lipid bilayers, comprising flat disks of lipids with both sides surrounded by the same aqueous environment. They are widely used in structural/atomistic analyses of membrane binding proteins. On the other hand, three main methods are currently used to study protein structure: X-ray crystallography, cryo-electron microscopy and NMR (see Lexicon and Figure 3A). NMR is the only method that enables the studies at the atomistic level to be conducted in the solid state as well as in solution. NMR makes it possible to investigate protein dynamics, as well as structure, which gives access to the more intimate biological mechanisms of proteins. To gain an insight into the structure of the MA domain of HIV-1 Gag in interaction with the membrane, NMR experiments were initially performed in solution with di-C4 and di-C8 PI(4,5)P_2_ lipids complexed to MA [5]. From these experiments, a structure was proposed, exhibiting a strong interaction of the highly basic region (HBR) of MA with the polar head of the PI(4,5)P_2_, a switch of the myristoyl group of the MA from an hydrophobic pocket of the protein towards the exterior, supposedly to the PM and, at the same time, a swap of the 2′ acyl chain of the diC8-PI(4,5)P_2_ into another hydrophobic pocket of the MA. However, since the NMR was performed in solution, these structural rearrangements could be questioned [36]. Subsequently, Vlach and Saad complemented their structural characterization by using diC8-PI(4,5)P_2_ in addition to different diC6 acylated lipids (PhosphatidylCholine (PC), PhosphatidylEthanoamine (PE) and PhosphatidylSerine(PS)) [37]. They observed that not only did the 2′acyl chain of the PI(4,5)P_2_ flip into an MA hydrophobic pocket but that, moreover, in the case of PC, PS or PE, their 2′ acyl chains flipped into distinct MA hydrophobic pockets. More recently, thanks to the use of bicelles, Mercredi et al. performed NMR and revisited these previously established MA structures on membranes [38]. While they observed a strong interaction of the MA HBR with the polar head of the PI(4,5)P_2_ and, to a lesser extent, an interaction with the PS head at the MA position identified by [37], they did not observe any flipping of the acyl chains of these lipids into any of the two hydrophobic pockets. This was previously suggested by a coarse-grained molecular dynamic model of HIV-1 MA on PI(4,5)P_2_/PS membranes [39]. The added value of bicelles for NMR structure elucidation is clearly shown by this example, in which, when isolated, the phopholipid does not interact with the MA in the same way as when it is inserted into its natural environment, i.e., an amphiphilic membrane.

Wang and Huong also used bicelles to investigate the membrane curvature induction by IAV M2 protein (a transmembrane viral channel that is important for virus entry and particle budding) using OMAS-NMR [40] (see Lexicon). They showed that the M2 amphipathic helix (M2 AH), along with its transmembrane domain (M2 TM), induced strong membrane curvature in the bicelles, which in turn favored and stabilized the localization of the M2 in these strongly curved domains. These findings highlight the functional role of M2 during IAV assembly and is involvement in membrane deformation to promote IAV particle budding (reviewed in [9]).

### 3.3. Unilamellar Vesicles

As they are easy to make, and their composition is easy to tune, large unilamellar vesicles (LUVs) have been widely used in the field of viral assembly. 

Using LUVs, many different mechanisms of the HIV-1 Gag interaction with membranes were determined (Figure 3B). The residues of the matrix were identified as specific to the PI(4,5)P_2_ interactions [41,42]. Preferential lipid compositions were tested in order to monitor the effect of the different lipids [43,44,45] and the main role of PI(4,5)P_2_ was confirmed [46]. The roles of myristoylation and the electrostatic nature of the interaction were also identified and quantified using LUVs [42,47,48]. The binding equilibrium of the MA domain of the Gag between the membrane lipids and the RNA was also investigated thanks to LUVs. For example, Chukkapalli et al. highlighted the importance of five residues found to bind RNA, which also restricts MA binding to membranes lacking PI(4,5)P_2_, supporting the role of RNA in masking the non-specific binding of Gag to membranes [49] and the regulation of membrane binding by t-RNA [50,51]. 

LUVs also help to understand the lipid organization/composition in the membrane that rules the assembly. For example, self-assembling matrix lipid-phase partitioning and lipid phase separation occurring during Gag self-assembly were investigated with LUVs [44,52]. Recently, Urbančič et al. [53] determined that lipid composition but not membrane curvature influenced HIV envelope-like lipid membrane fluidity by measuring the lipid diffusion through line-scanning stimulated emission depletion-fluorescence correlation spectroscopy (STED-FCS) (see Lexicon).

LUVs have heavily contributed to the understanding of HIV-1 Gag membrane interactions initiating the generation of new viruses, but they are also key tools to decipher IAV matrix protein/lipid membrane interactions.

Since the pioneering work of Gregoriades and Oxford [54,55], PS has been identified as the main target for the membrane association of IAV M1 [16,56,57]. Recently, the effect of lipid composition on the initiation of M1 self-assembly has been studied using SUVs (small unilamellar vesicles) and proteoliposomes, showing the role of lipid ordering in the association of M1 to PS in these liposomes [58]. Liposomes were also used to monitor the interactions of virus proteins such as HA [55] and NA [59] with lipids and lipid redistribution; however, this was essentially considered during membrane fusion [60], which is the entry step (early phase of replication) of the virus, not the assembly step. A significant effort has been directed at the understanding of the M2 protein’s interaction with lipids in the case of IAV. This because M2 is a transmembrane protein and is described as playing a major role in the budding of the Influenza virus by inducing membrane scission [61]. Virus assembly and budding generate changes in the local curvatures of membranes. At the end of assembly, a neck appears at the bud with a strong and unstable local curvature. In the case of IAV, M2, thanks to its amphipatic helix (AH), has been proposed to both sense and stabilize this local curvature [40]. This localization has been confirmed both by using liposomes of different curvatures [62] and by using liposomes containing different cholesterol concentrations, showing that cholesterol induces the reorientation of this amphipatic helix [63]. 

Ebola virus assembly is poorly studied with membrane model systems. However, 20 years ago, VP40 structure and hexamerization were studied using liposomes [64,65]. The C-terminal truncation of the VP40 protein was shown to be responsible for its spontaneous hexamerization, suggesting its main role in the initiation step of EBOV assembly. Scianimanico et al. observed, using the membrane flotation technique (see Lexicon), that VP40 membrane association triggered hexamerization [65]. Using combination of surface plasmon resonance (SPR) (see Lexicon) and membrane flotation assays, the membrane binding of VP40 was shown to be essentially mediated by its interaction with PS [35] and PI(4,5)P_2_ [18]; this was also confirmed in living cells in the same studies. 

### 3.4. Supported and Tethered Bilayers: Planar Membranes

SLBs are planar lipid membranes lying on the surfaces of glass coverslips. One major advantage is that they offer a widely accessible environment in which to study protein binding, diffusion and the lipid-induced reorganization occurring simultaneously. A second advantage is the possibility of using a wide object only a few nm in height, allowing its direct observation at the molecular level by using techniques such as atomic force microscopy (AFM) (see Lexicon and Figure 3C). For these reasons, SLBs were used to study viral protein assembly using AFM [66]. 

Using SLBs with a lipid composition mimicking the inner leaflet of the cell plasma membrane, as well as measuring the self-quenching of fluorescent lipids, Yandrapalli et al. showed that, upon self-assembly, HIV-1 Gag generated PI(4,5)P_2_/Cholesterol clusters [44]. This finding questioned the model suggesting that either HIV-1 Gag targeted the pre-enriched lipid domains of the membrane to self-assemble or generated its own lipid bed. Interestingly, in a subsequent study, this PI(4,5)P_2_/Cholesterol nano-clustering was also shown to occur during the assembly of new HIV-1 viruses at the host plasma membrane of HIV-1-infected T cells [67], revealing that HIV-1 Gag self-assembly is the driving force in PI(4,5)P_2_/Cholesterol-enriched lipid nanodomain generation. 

It is commonly accepted that Gag-Gag interactions are sufficient to produce the energy required to initiate HIV-1 assembly. However, using atomic force microscopy (AFM) on SLBs, Miles et al. observed that interactions between HIV-1 envelope glycoproteins Gp41 and Gp120 could also partially drive viral assembly [68]. The SLBs were processed by vesicle fusion and Gp41 containing vesicles were fused to the SLBs. The Gp120 proteins were then consecutively injected and formed wire-shaped structures at the bilayer surface. The described interactions between these Gp proteins, although they were weaker and formed abnormally shaped assembling particles, could, according to the authors, be considered as a driving force in viral assembly.

S. Chiantia’s group intensively used SLBs to explore IAV assembly by monitoring M1-M1 interactions with quantitative optical microscopy. Using RICS (see Lexicon) and FCS, in addition to AFM (Figure 3C), Hilsch et al. described M1 self-assembly enhancement in PS-containing membranes [15]. They proposed that M1 self-assembly was sufficient to initiate the formation of new viral particles, even in the absence of other viral proteins. 

These results were confirmed subsequently and the interplay between PS and M1, in which M1 interacts mostly with PS-enriched domains within lipid bilayers and stabilizes these PS domains during M1 self-assembly, was described in detail [69].

Finally, thanks to RICS, SPR and circular dichroism spectroscopy (see Lexicon), a subsequent investigation of the precise mechanism of the interaction between IAV M1 protein and the PS-enriched bilayer was published by Höfer et al. in 2019 [70]. A specific conformational change was found to occur upon the M1’s binding to the negatively charged PS. From the structures in the protein data bank (PDB) and simulations, N-terminal domains of M1 was shown to be involved in membrane binding, stabilizing C-terminal domain to favor self-assembly.

SLBs are standard models for lipid diffusion measurements. However, a two-to-threefold decrease in lipid diffusion coefficients is usually measured between SLBs and liposomes or GUVs. Indeed, Van der Waals interactions between the glass substrate and the lipids monolayer in contact with it led to an overall decrease in diffusion [71]. This decrease in lipid mobility in one monolayer could impede the lateral organization of lipids and therefore play an artificial role in viral protein membrane binding. To remove these Van der Waals interactions, a sparsely tethered bilayer lipid membrane (stBLM) system was used by Barros et al. to quantify, using SPR, HIV-1 Gag MA binding to the membrane. They showed that MA was attracted to the membrane by charged lipids, while the MA myristate exposure increased the membrane affinity 100-fold. They also highlighted that cholesterol facilitated myristate insertion and PI(4,5)P_2_ binding without interacting with the MA. Interestingly, the concomitant role of cholesterol and PI(4,5)P_2_ was also observed in [44] using classical SLBs, suggesting again that the lipid organization for correct binding and virus formation is mainly driven by Gag-Gag self-assembly and not by pre-existing lipid domains.

### 3.5. Giant Unilamellar Vesicles: The Closest Model to Cell Plasma Membranes

Amongst all the models used to mimic cellular membranes, GUVs have the closest properties to the cell plasma membrane. They are spherical lipid bilayers with a diameter range equivalent to that of a eukaryotic cell (~10 µm) enclosing and surrounded by an aqueous medium. Their similarities with eukaryotic cells make it possible to study, in GUVs, several molecular properties of membrane lipids and their interactions with viral proteins, such as lipid ordering, lipid–protein interaction localization, and curvature or tubular structure induction.

GUVs were used models to directly visualize HIV-1 Gag proteins’ binding properties and localization in membranes with complex lipid compositions. When preparing GUVs with two separate phases, i.e., liquid-ordered and liquid-disordered, it was possible to show that the Gag, as well as a the multimerizing MA domain of the Gag, mainly partitioned into liquid-disordered phase where PI(4,5)P_2_ was present and that this partitioning did not change upon Gag self-assembly [44,52] (Figure 3D). These results were confirmed recently using the same approach [72].

Another interesting point regarding GUVs is that their wide, flat lipid surfaces make it possible to study not only protein assembly initiation but also aspects of its termination, such as viral budding. Gui et al. developed a protocol using a GUV model without cellular proteins that makes it possible to infer that Gag proteins’ self-aggregation alone leads to vesicle formation budding from the GUV membrane (an indirect observation based on GUV size-reduction measurements) [73]. However, in the late steps of retroviral assembly, the cellular endosomal sorting complex (ESCRT) machinery is recruited by Gag-p6 domain interacting with tumor susceptibility gene 101 (Tsg101) at HIV-1 lattices in order to facilitate virus budding [74]. Subsequently, using GUVs, Carlson and Hurley reconstituted an in vitro minimal system to monitor the dynamics of ESCRT machinery recruitment at HIV-1 Gag budding sites [75].

GUVs have also been extensively used to decipher Influenza virus assembly. Dahmani et al. observed, using confocal microscopy, that M1 binding to PS loaded GUVs induced the local deformation of the lipidic membrane [76]. By monitoring spatial changes in M1 dynamics through scanning FCS, they measured a decrease in M1 mobility at the location where membrane curvature was modified and concluded that solely M1-M1 interactions are sufficient to generate lipid membrane curvature. Similar behavior was observed with another strain of Influenza virus (namely, Influenza C Virus), where M1 proteins were able to induce tubular structures on GUVs [77]. 

In the case of Ebola virus, GUVs were also used to elucidate the interaction of EBOV matrix VP40 with lipids, showing the capacity of VP40 to penetrate into the lipid membrane [78] and to selectively induce vesicles after self-assembly on PS-enriched domains [79].

## 4. Conclusions

This review reveals the important role played by model membranes during the last 30 years in deciphering the initiation and elongation of the viral assembly of enveloped viruses on membranes. Several molecular mechanisms were made possible to assess by biomimetic membranes reconstituted in vitro. These results suggest that viral matrix proteins’ induction of membrane deformation upon the recognition of charged phospholipids on membranes is a common feature in the assembly of enveloped viruses. Our review focused on lethal human viruses assembling and budding from the plasma membrane of their host cells. Overall, we showed that biomimetic lipid membranes are very convenient models with which to study viral proteins’ surface oligomerization. Nevertheless, different limitations can be reported. Although these are very basic, difficulties occur when studying the transmembrane proteins involved in the assembly process simply because it is always difficult to correctly insert transmembrane proteins inside the lipid bilayer (while keeping the orientation, structure and functionality of the protein), especially when these form channels made of several subunits (such as Influenza’s M2 ionic channel). Limitations are also inherent in their own definitions. Model membranes cannot capture all the complexity of natural cellular membranes, not only in terms of protein and lipid composition diversity, but also because structural components tightly linked to the membrane functions and mechanical properties, such as glycocalyx and the inner cortical actin cytoskeleton, are absent. To overcome this, recently, methods have been developed to generate giant plasma membrane vesicles (GPMVs) derived from host-cell PMs, which conserve cell PM complexity and organization, but without the inner cytoskeleton [80,81,82]. However, it will certainly be helpful to shed light on the interplay between viral and host-cell molecular membrane components during viral assembly.

Since the emergence of new viruses is predicted to increase due to global warming, there is no doubt that, in the near future, model membranes will continue to play a role in deciphering the molecular mechanisms of virus assemblies and will certainly be useful for the in vitro testing of new antiviral drugs targeting viral protein assembly on membranes. 

## Data Availability

Not applicable.

## Glossary

**Biophysical Techniques Lexicon**		
**HX MS**	*Hydrogen Exchange Mass Spectrometry*	This technique exploits proteins’ fundamental ability to exchange hydrogens with their environment. Studied proteins are incorporated in deuterated water (D_2_O) and exchange hydrogens bonded to the backbone nitrogen with deuterium from D_2_O solvent over time. Incorporated deuterium atoms are consecutively used to probe protein conformation using mass spectrometry [83].
	*X-ray Crystallography*	The diffraction of incident X-rays by electrons in a sample is used to calculate the positions of atoms in molecules. Due to the fact that the signal of each individual atom is weak, this technique requires sample crystallization (all the molecules are immobilized in the same conformation in a 3D lattice) [84].
**Cryo-EM**	*Cryo-electron microscopy*	This process involves freezing samples at cryogenic temperatures so quickly that water molecules do not crystallize, which preserves the samples’ native structure. This sample processing technique is generally associated with transmission electron microscopy for structural analyses at an atomic level [85].
**NMR**	*Nuclear Magnetic Resonance*	NMR is based on the spin properties of atomic nuclei, which are aligned when submitted to a magnetic field. Only a few isotopes that have a magnetic spin are used for NMR purposes (the most common is ^1^H, but ^13^C, ^15^N and ^31^P are also often used), and the analysis either relies on the natural isotopic proportion in the sample or is performed on enriched samples. Once irradiated, each non-equivalent nucleus resonates at a specific frequency, making peaks in the NMR signal, called chemical shifts. The peaks’ intensity and multiplicity are used to determine the atoms’ spatial proximity [86].
**OMAS-NMR**	*Off-Magic-Angle Spinning NMR*	This is a variant of the magic-angle-pinning solid-state NMR developed to narrow the quadrupolar resonances and increase the frequency resolution/attribution. Resonance widening occurs in anisotropic systems, such as proteins bound to lipid membranes. OMAS is based on a slight deviation from the magic angle (angle < 0.01°). This significantly improves the precision and accuracy of derived rate parameters, especially for slow motion on the kilohertz time scale [87].
**FCS**	*Fluorescence Correlation Spectroscopy* (STED, scanning, Spot Variation)	This microscopy technique relies on the use of fluorescently labelled particles and makes it possible to quantify their dynamics (including their concentration, diffusion coefficient and interactions) by recording and correlating the fluorescence intensity’s fluctuation over time in a given zone [88]. Using an optical microscope, a scanning mode can be added to perform scanning-FCS. An adjustable pinhole enables spot-variation-FCS. Coupled with a STED microscope, the reduced volume of detection makes it possible to study diffusion in nanoscale regions [89,90,91].
**STED**	*Stimulated Emission Depletion*	This is a super-resolution fluorescence microscopy technique based on the possibility to deplete the excited fluorophore before it emits. For this, a depletion laser (emitting at a higher wavelength than the excitation wavelength) is focused on the object plane, as a a donut shape surrounding the excitation laser beam. The fluorophore exposed to this donut shape laser returns to the ground state before emitting a fluorescence photon. This result in a continuous decreasing size of the emission spot below the diffraction limit (<200 nm)with increasing depletion laser power [92].
	*Liposome flotation assay*	This method is used to characterize the interactions between proteins and membrane-mimicking liposomes (LUV). This assay is based on bound/free proteins’ separation by centrifuging the complex through a sucrose gradient. At the appropriate centrifugation speed, proteins bound to liposomes float up to the low-sucrose fraction, whereas denser free proteins remain at the bottom at high sucrose-density levels. Due to protein–LUV interactions, an apparent K_D_ value for the liposome–protein interaction can be estimated by varying the amount of LUV at a constant protein concentration [93,94].
**SPR**	*Surface Plasmon Resonance*	SPR describes a propagation phenomenon of electrons parallel to a metal layer when excited by an incident laser at a certain angle (with the angle depending on the refractive index of the material near the metal surface). An SPR assay is based on the lowered SPR occurrence due to an analyte interaction with its target, coated at the surface of the metal layer. The amount of interacting analytes and the interaction kinetics are unveiled either by measuring the reflected light intensity or by tracking the resonance angle shift [95].
**RICS**	*Raster Image Correlation Spectroscopy*	Raster image correlation spectroscopy (RICS) is a fluorescent-image-analysis method for extracting the mobility, concentration and stoichiometry of diffusing fluorescent molecules from confocal image stacks. The method works by calculating a spatial correlation function for each image and analysing their average by model fitting [96].
**CD Spectroscopy**	*Circular Dichroism Spectroscopy*	Polarized absorption spectroscopy method. When illuminated by an elliptical polarized light, the absorption of the incident light will depend on chirality, but also on the folding of the structures in the case of complex macromolecules. It is generally used to characterize the conformation of a protein’s secondary structure and, in some cases, of its tertiary structure environment. It also enables access to dynamic conformational changes in proteins in or at membranes [97].

## References

[1] Bernardino de la Serna J., Schütz G.J., Eggeling C., Cebecauer M. (2016). There Is No Simple Model of the Plasma Membrane Organization. Front. Cell Dev. Biol..

[2] Jouvenet N., Bieniasz P.D., Simon S.M. (2008). Imaging the Biogenesis of Individual HIV-1 Virions in Live Cells. Nature.

[3] Floderer C., Masson J.-B., Boilley E., Georgeault S., Merida P., El Beheiry M., Dahan M., Roingeard P., Sibarita J.-B., Favard C. (2018). Single Molecule Localisation Microscopy Reveals How HIV-1 Gag Proteins Sense Membrane Virus Assembly Sites in Living Host CD4 T Cells. Sci. Rep..

[4] Michaels T.C.T., Bellaiche M.M.J., Hagan M.F., Knowles T.P.J. (2017). Kinetic Constraints on Self-Assembly into Closed Supramolecular Structures. Sci. Rep..

[5] Saad J.S., Miller J., Tai J., Kim A., Ghanam R.H., Summers M.F. (2006). Structural Basis for Targeting HIV-1 Gag Proteins to the Plasma Membrane for Virus Assembly. Proc. Natl. Acad. Sci. USA.

[6] Saad J.S., Muriaux D.M. (2015). Editorial: Role of Lipids in Virus Assembly. Front. Microbiol..

[7] Muriaux D., Darlix J.-L. (2010). Properties and Functions of the Nucleocapsid Protein in Virus Assembly. RNA Biol..

[8] Kutluay S.B., Bieniasz P.D. (2010). Analysis of the Initiating Events in HIV-1 Particle Assembly and Genome Packaging. PLoS Pathog..

[9] Rossman J.S., Lamb R.A. (2011). Influenza Virus Assembly and Budding. Virology.

[10] Motsa B.B., Stahelin R.V. (2021). Lipid–Protein Interactions in Virus Assembly and Budding from the Host Cell Plasma Membrane. Biochem. Soc. Trans..

[11] Panahandeh S., Li S., Marichal L., Leite Rubim R., Tresset G., Zandi R. (2020). How a Virus Circumvents Energy Barriers to Form Symmetric Shells. ACS Nano.

[12] Nguyen T.T., Shklovskii B.I. (2002). Kinetics of Macroion Coagulation Induced by Multivalent Counterions. Phys. Rev. E.

[13] Hogue I.B., Hoppe A., Ono A. (2009). Quantitative Fluorescence Resonance Energy Transfer Microscopy Analysis of the Human Immunodeficiency Virus Type 1 Gag-Gag Interaction: Relative Contributions of the CA and NC Domains and Membrane Binding. J. Virol..

[14] Gamble T.R., Yoo S., Vajdos F.F., von Schwedler U.K., Worthylake D.K., Wang H., McCutcheon J.P., Sundquist W.I., Hill C.P. (1997). Structure of the Carboxyl-Terminal Dimerization Domain of the HIV-1 Capsid Protein. Science.

[15] Hilsch M., Goldenbogen B., Sieben C., Höfer C.T., Rabe J.P., Klipp E., Herrmann A., Chiantia S. (2014). Influenza A Matrix Protein M1 Multimerizes upon Binding to Lipid Membranes. Biophys. J..

[16] Kerviel A., Dash S., Moncorgé O., Panthu B., Prchal J., Décimo D., Ohlmann T., Lina B., Favard C., Decroly E. (2016). Involvement of an Arginine Triplet in M1 Matrix Protein Interaction with Membranes and in M1 Recruitment into Virus-Like Particles of the Influenza A(H1N1)Pdm09 Virus. PLoS ONE.

[17] Petrich A., Dunsing V., Bobone S., Chiantia S. (2021). Influenza A M2 Recruits M1 to the Plasma Membrane: A Fluorescence Fluctuation Microscopy Study. Biophys. J..

[18] Johnson K.A., Taghon G.J.F., Scott J.L., Stahelin R.V. (2016). The Ebola Virus Matrix Protein, VP40, Requires Phosphatidylinositol 4,5-Bisphosphate (PI(4,5)P2) for Extensive Oligomerization at the Plasma Membrane and Viral Egress. Sci. Rep..

[19] Kielian M., Rey F.A. (2006). Virus Membrane-Fusion Proteins: More than One Way to Make a Hairpin. Nat. Rev. Microbiol..

[20] Más V., Melero J.A., Mateu M.G. (2013). Entry of Enveloped Viruses into Host Cells: Membrane Fusion. Structure and Physics of Viruses.

[21] Mouritsen O.G. (2011). Model Answers to Lipid Membrane Questions. Cold Spring Harb. Perspect. Biol..

[22] Zasadzinski J.A., Viswanathan R., Madsen L., Garnaes J., Schwartz D.K. (1994). Langmuir-Blodgett Films. Science.

[23] Sanders C.R., Prosser R.S. (1998). Bicelles: A Model Membrane System for All Seasons?. Structure.

[24] Hutchison J.M., Shih K.-C., Scheidt H.A., Fantin S.M., Parson K.F., Pantelopulos G.A., Harrington H.R., Mittendorf K.F., Qian S., Stein R.A. (2020). Bicelles Rich in Both Sphingolipids and Cholesterol and Their Use in Studies of Membrane Proteins. J. Am. Chem. Soc..

[25] Schwendener R.A., Chan W.C.W. (2007). Liposomes in Biology and Medicine. Bio-Applications of Nanoparticles.

[26] Tamm L.K., McConnell H.M. (1985). Supported Phospholipid Bilayers. Biophys. J..

[27] Veneziano R., Rossi C., Chenal A., Brenner C., Ladant D., Chopineau J. (2017). Synthesis and Characterization of Tethered Lipid Assemblies for Membrane Protein Reconstitution (Review). Biointerphases.

[28] Reeves J.P., Dowben R.M. (1969). Formation and Properties of Thin-Walled Phospholipid Vesicles. J. Cell. Physiol..

[29] Weinberger A., Tsai F.-C., Koenderink G.H., Schmidt T.F., Itri R., Meier W., Schmatko T., Schröder A., Marques C. (2013). Gel-Assisted Formation of Giant Unilamellar Vesicles. Biophys. J..

[30] Angelova M.I., Dimitrov D.S. (1986). Liposome Electroformation. Faraday Discuss. Chem. Soc..

[31] Chiantia S., Schwille P., Klymchenko A.S., London E. (2011). Asymmetric GUVs Prepared by MβCD-Mediated Lipid Exchange: An FCS Study. Biophys. J..

[32] Enoki T.A., Wu J., Heberle F.A., Feigenson G.W. (2021). Investigation of the Domain Line Tension in Asymmetric Vesicles Prepared via Hemifusion. Biochim. Biophys. Acta Biomembr..

[33] Malbec M., Sourisseau M., Guivel-Benhassine F., Porrot F., Blanchet F., Schwartz O., Casartelli N. (2013). HIV-1 Nef Promotes the Localization of Gag to the Cell Membrane and Facilitates Viral Cell-to-Cell Transfer. Retrovirology.

[34] Pirrone G.F., Emert-Sedlak L.A., Wales T.E., Smithgall T.E., Kent M.S., Engen J.R. (2015). Membrane-Associated Conformation of HIV-1 Nef Investigated with Hydrogen Exchange Mass Spectrometry at a Langmuir Monolayer. Anal. Chem..

[35] Adu-Gyamfi E., Soni S.P., Xue Y., Digman M.A., Gratton E., Stahelin R.V. (2013). The Ebola Virus Matrix Protein Penetrates into the Plasma Membrane. J. Biol. Chem..

[36] Kerviel A., Thomas A., Chaloin L., Favard C., Muriaux D. (2013). Virus Assembly and Plasma Membrane Domains: Which Came First?. Virus Res..

[37] Vlach J., Saad J.S. (2013). Trio Engagement via Plasma Membrane Phospholipids and the Myristoyl Moiety Governs HIV-1 Matrix Binding to Bilayers. Proc. Natl. Acad. Sci. USA.

[38] Mercredi P.Y., Bucca N., Loeliger B., Gaines C.R., Mehta M., Bhargava P., Tedbury P.R., Charlier L., Floquet N., Muriaux D. (2016). Structural and Molecular Determinants of Membrane Binding by the HIV-1 Matrix Protein. J. Mol. Biol..

[39] Charlier L., Louet M., Chaloin L., Fuchs P., Martinez J., Muriaux D., Favard C., Floquet N. (2014). Coarse-Grained Simulations of the HIV-1 Matrix Protein Anchoring: Revisiting Its Assembly on Membrane Domains. Biophys. J..

[40] Wang T., Hong M. (2015). Investigation of the Curvature Induction and Membrane Localization of the Influenza Virus M2 Protein Using Static and Off-Magic-Angle Spinning Solid-State Nuclear Magnetic Resonance of Oriented Bicelles. Biochemistry.

[41] Zhou W., Resh M.D. (1996). Differential Membrane Binding of the Human Immunodeficiency Virus Type 1 Matrix Protein. J. Virol..

[42] Dalton A.K., Ako-Adjei D., Murray P.S., Murray D., Vogt V.M. (2007). Electrostatic Interactions Drive Membrane Association of the Human Immunodeficiency Virus Type 1 Gag MA Domain. J. Virol..

[43] Ehrlich L.S., Fong S., Scarlata S., Zybarth G., Carter C. (1996). Partitioning of HIV-1 Gag and Gag-Related Proteins to Membranes. Biochemistry.

[44] Yandrapalli N., Lubart Q., Tanwar H.S., Picart C., Mak J., Muriaux D., Favard C. (2016). Self Assembly of HIV-1 Gag Protein on Lipid Membranes Generates PI(4,5)P2/Cholesterol Nanoclusters. Sci. Rep..

[45] Dick R.A., Goh S.L., Feigenson G.W., Vogt V.M. (2012). HIV-1 Gag Protein Can Sense the Cholesterol and Acyl Chain Environment in Model Membranes. Proc. Natl. Acad. Sci. USA.

[46] Chukkapalli V., Hogue I.B., Boyko V., Hu W.-S., Ono A. (2008). Interaction between the Human Immunodeficiency Virus Type 1 Gag Matrix Domain and Phosphatidylinositol-(4,5)-Bisphosphate Is Essential for Efficient Gag Membrane Binding. J. Virol..

[47] Bouamr F., Scarlata S., Carter C. (2003). Role of Myristylation in HIV-1 Gag Assembly. Biochemistry.

[48] Pérez Socas L.B., Ambroggio E.E. (2020). The Influence of Myristoylation, Liposome Surface Charge and Nucleic Acid Interaction in the Partition Properties of HIV-1 Gag-N-Terminal Peptides to Membranes. Biochim. Biophys. Acta Biomembr..

[49] Chukkapalli V., Oh S.J., Ono A. (2010). Opposing Mechanisms Involving RNA and Lipids Regulate HIV-1 Gag Membrane Binding through the Highly Basic Region of the Matrix Domain. Proc. Natl. Acad. Sci. USA.

[50] Sumner C., Kotani O., Liu S., Musier-Forsyth K., Sato H., Ono A. (2022). Molecular Determinants in TRNA D-Arm Required for Inhibition of HIV-1 Gag Membrane Binding. J. Mol. Biol..

[51] Todd G.C., Duchon A., Inlora J., Olson E.D., Musier-Forsyth K., Ono A. (2017). Inhibition of HIV-1 Gag–Membrane Interactions by Specific RNAs. RNA.

[52] Keller H., Kräusslich H.-G., Schwille P. (2013). Multimerizable HIV Gag Derivative Binds to the Liquid-Disordered Phase in Model Membranes. Cell. Microbiol..

[53] Urbančič I., Brun J., Shrestha D., Waithe D., Eggeling C., Chojnacki J. (2018). Lipid Composition but Not Curvature Is the Determinant Factor for the Low Molecular Mobility Observed on the Membrane of Virus-Like Vesicles. Viruses.

[54] Gregoriades A. (1980). Interaction of Influenza M Protein with Viral Lipid and Phosphatidylcholine Vesicles. J. Virol..

[55] Oxford J.S., Hockley D.J., Heath T.D., Patterson S. (1981). The Interaction of Influenza Virus Haemagglutinin with Phospholipid Vesicles—Morphological and Immunological Studies. J. Gen. Virol..

[56] Baudin F., Petit I., Weissenhorn W., Ruigrok R.W. (2001). In Vitro Dissection of the Membrane and RNP Binding Activities of Influenza Virus M1 Protein. Virology.

[57] Ruigrok R.W., Barge A., Durrer P., Brunner J., Ma K., Whittaker G.R. (2000). Membrane Interaction of Influenza Virus M1 Protein. Virology.

[58] Kordyukova L.V., Konarev P.V., Fedorova N.V., Shtykova E.V., Ksenofontov A.L., Loshkarev N.A., Dadinova L.A., Timofeeva T.A., Abramchuk S.S., Moiseenko A.V. (2021). The Cytoplasmic Tail of Influenza A Virus Hemagglutinin and Membrane Lipid Composition Change the Mode of M1 Protein Association with the Lipid Bilayer. Membranes.

[59] Huang R.T.C., Warn K., Klenk H.-D., Rott R. (1979). Association of the Envelope Glycoproteins of Influenza Virus with Liposomes—a Model Study on Viral Envelope Assembly. Virology.

[60] Bailey A., Zhukovsky M., Gliozzi A., Chernomordik L.V. (2005). Liposome Composition Effects on Lipid Mixing between Cells Expressing Influenza Virus Hemagglutinin and Bound Liposomes. Arch. Biochem. Biophys..

[61] Rossman J.S., Jing X., Leser G.P., Lamb R.A. (2010). Influenza Virus M2 Protein Mediates ESCRT-Independent Membrane Scission. Cell.

[62] Martyna A., Gómez-Llobregat J., Lindén M., Rossman J.S. (2016). Curvature Sensing by a Viral Scission Protein. Biochemistry.

[63] Martyna A., Bahsoun B., Madsen J.J., Jackson F.S.J.S., Badham M.D., Voth G.A., Rossman J.S. (2020). Cholesterol Alters the Orientation and Activity of the Influenza Virus M2 Amphipathic Helix in the Membrane. J. Phys. Chem. B.

[64] Ruigrok R.W.H., Schoehn G., Dessen A., Forest E., Volchkov V., Dolnik O., Klenk H.-D., Weissenhorn W. (2000). Structural Characterization and Membrane Binding Properties of the Matrix Protein VP40 of Ebola Virus11Edited by J. Karn. J. Mol. Biol..

[65] Scianimanico S. (2000). Membrane Association Induces a Conformational Change in the Ebola Virus Matrix Protein. EMBO J..

[66] Valbuena A., Maity S., Mateu M.G., Roos W.H. (2020). Visualization of Single Molecules Building a Viral Capsid Protein Lattice through Stochastic Pathways. ACS Nano.

[67] Favard C., Chojnacki J., Merida P., Yandrapalli N., Mak J., Eggeling C., Muriaux D. (2019). HIV-1 Gag Specifically Restricts PI(4,5)P2 and Cholesterol Mobility in Living Cells Creating a Nanodomain Platform for Virus Assembly. Sci. Adv..

[68] Miles P., Cassidy P., Donlon L., Yarkoni O., Frankel D. (2015). In Vitro Assembly of a Viral Envelope. Soft Matter.

[69] Bobone S., Hilsch M., Storm J., Dunsing V., Herrmann A., Chiantia S. (2017). Phosphatidylserine Lateral Organization Influences the Interaction of Influenza Virus Matrix Protein 1 with Lipid Membranes. J. Virol..

[70] Höfer C.T., Di Lella S., Dahmani I., Jungnick N., Bordag N., Bobone S., Huang Q., Keller S., Herrmann A., Chiantia S. (2019). Structural Determinants of the Interaction between Influenza A Virus Matrix Protein M1 and Lipid Membranes. Biochim. Biophys. Acta Biomembr..

[71] Cremer P.S., Boxer S.G. (1999). Formation and Spreading of Lipid Bilayers on Planar Glass Supports. J. Phys. Chem. B.

[72] Wen Y., Feigenson G.W., Vogt V.M., Dick R.A. (2020). Mechanisms of PI(4,5)P2 Enrichment in HIV-1 Viral Membranes. J. Mol. Biol..

[73] Gui D., Gupta S., Xu J., Zandi R., Gill S., Huang I.-C., Rao A.L.N., Mohideen U. (2015). A Novel Minimal in Vitro System for Analyzing HIV-1 Gag-Mediated Budding. J. Biol. Phys..

[74] Van Engelenburg S.B., Shtengel G., Sengupta P., Waki K., Jarnik M., Ablan S.D., Freed E.O., Hess H.F., Lippincott-Schwartz J. (2014). Distribution of ESCRT Machinery at HIV Assembly Sites Reveals Virus Scaffolding of ESCRT Subunits. Science.

[75] Carlson L.-A., Hurley J.H. (2012). In Vitro Reconstitution of the Ordered Assembly of the Endosomal Sorting Complex Required for Transport at Membrane-Bound HIV-1 Gag Clusters. Proc. Natl. Acad. Sci. USA.

[76] Dahmani I., Ludwig K., Chiantia S. (2019). Influenza A Matrix Protein M1 Induces Lipid Membrane Deformation via Protein Multimerization. Biosci. Rep..

[77] Saletti D., Radzimanowski J., Effantin G., Midtvedt D., Mangenot S., Weissenhorn W., Bassereau P., Bally M. (2017). The Matrix Protein M1 from Influenza C Virus Induces Tubular Membrane Invaginations in an in Vitro Cell Membrane Model. Sci. Rep..

[78] Soni S.P., Adu-Gyamfi E., Yong S.S., Jee C.S., Stahelin R.V. (2013). The Ebola Virus Matrix Protein Deeply Penetrates the Plasma Membrane: An Important Step in Viral Egress. Biophys. J..

[79] Soni S.P., Stahelin R.V. (2014). The Ebola Virus Matrix Protein VP40 Selectively Induces Vesiculation from Phosphatidylserine-Enriched Membranes. J. Biol. Chem..

[80] Podkalicka J., Blouin C.M., Blouin C.M. (2020). GPMVs as a Tool to Study Caveolin-Interacting Partners. Caveolae.

[81] Levental K.R., Levental I. (2015). Giant Plasma Membrane Vesicles: Models for Understanding Membrane Organization. Current Topics in Membranes.

[82] Gerstle Z., Desai R., Veatch S.L. (2018). Giant Plasma Membrane Vesicles: An Experimental Tool for Probing the Effects of Drugs and Other Conditions on Membrane Domain Stability. Methods in Enzymology.

[83] Marcsisin S.R., Engen J.R. (2010). Hydrogen Exchange Mass Spectrometry: What Is It and What Can It Tell Us?. Anal. Bioanal. Chem..

[84] (2017). Molecular Virology of Human Pathogenic Viruses.

[85] Milne J.L.S., Borgnia M.J., Bartesaghi A., Tran E.E.H., Earl L.A., Schauder D.M., Lengyel J., Pierson J., Patwardhan A., Subramaniam S. (2013). Cryo-Electron Microscopy—A Primer for the Non-Microscopist. FEBS J..

[86] Howard M.J. (1998). Protein NMR Spectroscopy. Curr. Biol..

[87] Huang Y., Vold R.L., Hoatson G.L. (2006). Investigation of Multiaxis Molecular Motion by Off-Magic Angle Spinning Deuteron NMR. J. Chem. Phys..

[88] Magde D., Elson E., Webb W.W. (1972). Thermodynamic Fluctuations in a Reacting System—Measurement by Fluorescence Correlation Spectroscopy. Phys. Rev. Lett..

[89] Wawrezinieck L., Rigneault H., Marguet D., Lenne P.-F. (2005). Fluorescence Correlation Spectroscopy Diffusion Laws to Probe the Submicron Cell Membrane Organization. Biophys. J..

[90] Eggeling C., Ringemann C., Medda R., Schwarzmann G., Sandhoff K., Polyakova S., Belov V.N., Hein B., von Middendorff C., Schönle A. (2009). Direct Observation of the Nanoscale Dynamics of Membrane Lipids in a Living Cell. Nature.

[91] Favard C., Wenger J., Lenne P.-F., Rigneault H. (2011). FCS Diffusion Laws in Two-Phase Lipid Membranes: Determination of Domain Mean Size by Experiments and Monte Carlo Simulations. Biophys. J..

[92] Vicidomini G., Bianchini P., Diaspro A. (2018). STED Super-Resolved Microscopy. Nat. Methods.

[93] Zhao H., Lappalainen P. (2012). A Simple Guide to Biochemical Approaches for Analyzing Protein–Lipid Interactions. Mol. Biol. Cell.

[94] Hamard-Peron E., Juillard F., Saad J.S., Roy C., Roingeard P., Summers M.F., Darlix J.-L., Picart C., Muriaux D. (2010). Targeting of Murine Leukemia Virus Gag to the Plasma Membrane Is Mediated by PI(4,5)P2/PS and a Polybasic Region in the Matrix. J. Virol..

[95] (2019). Nano-Inspired Biosensors for Protein Assay with Clinical Applications.

[96] Digman M.A., Brown C.M., Sengupta P., Wiseman P.W., Horwitz A.R., Gratton E. (2005). Measuring Fast Dynamics in Solutions and Cells with a Laser Scanning Microscope. Biophys. J..

[97] Miles A.J., Janes R.W., Wallace B.A. (2021). Tools and Methods for Circular Dichroism Spectroscopy of Proteins: A Tutorial Review. Chem. Soc. Rev..

